# The technique and its role of dacryoendoscopy in the management of the false passage of the lacrimal drainage system

**DOI:** 10.1038/s41598-022-27135-5

**Published:** 2022-12-28

**Authors:** Myungjin Kim, Helen Lew

**Affiliations:** Department of Ophthalmology, CHA Bundang Medical Center, CHA University School of Medicine, 59, Yatap-ro, Bundang-gu, Seongnam-si, Gyeonggi-do 13496 Republic of Korea

**Keywords:** Anatomy, Signs and symptoms

## Abstract

This is a retrospective study of patients with primary acquired nasolacrimal duct obstruction (PANDO) who underwent dacryoendoscopy (FT-203F; Fibertech Co., Tokyo, Japan) and sheath-guided silicone intubation for 830 cases with PANDO from March 2016 to December 2020. 19 cases (2.3%) were observed as false passage in the lacrimal drainage system (LDS). Dacryoendoscopic findings revealed that the following factors were associated with LDS obstruction (% cases): structural change of 63.2% (stenosis, 42.1%; fibrotic membrane, 21.1%), and secretory change of 36.8% (mucus, 15.8%; stone, 10.5%; and granulation, 10.5%). The obstruction sites were distributed through LDS. The false passages detected in LDS were managed as follow: usage of fluid irrigation pressure to check the true passage following the previously intubated silicone tube as a reference, and confirmation the end of passage through the inferior meatus with nasal endoscopy. The overall success rate was 73.7% using this management technique. Dacryoendoscopy enables real-time observation of the lumen of the LDS, thus facilitating management of pathological lesions including false passages. With this technique, we are better able to make customized treatment of patients with false passages, with a safer and more effective results leading to the success of dacryoendoscopy guided silicone tube intubation in PANDO patients.

## Introduction

Nasolacrimal duct obstruction (NDO) means an obstruction of the lacrimal drainage system (LDS), which can manifest as epiphora or recurrent infections with mucopurulent discharge. It can occur at any level along the LDS, including the punctum, canaliculus, sac, nasolacrimal duct, or inferior meatus. It is important to understand the precise etiology and location of the obstruction to manage the NDO patients. Diagnostic methods for this condition include lacrimal irrigation, probing, dacryocystography (DCG), computed tomography-DCG, or echographic examination^1–3^. NDO patients with no apparent improvement of symptom despite the conservative treatments require surgical procedures. Surgical procedures for NDO patients are known as dacryocystorhinostomy (DCR) and silicone tube intubation (STI). Among them, STI is commonly used procedure because it has advantages such as short surgery time, less risk of bleeding during surgery, and fast recovery after surgery^4^.

The success rate of STI increased after the appearance of dacryoendoscopy which can investigate the passage from the punctum to the valve of Hasner^5^. According to previous studies about the clinical application of the dacryoendoscopy, Haefliger and Piffaretti^6^ used it for treatment of primary NDO patients and introduced it as a good method to remove lacrimal stone and fibrotic tissue on lacrimal duct during the surgery. Lee and Lew^7^ previously analyzed the lesions detected in DCG and dacryoendoscopy, and they found them corresponding in location or degree of occlusion in DCG and dacryoendoscopy. In other words, complementary use of DCG and dacryoendoscopy could be useful for patients with epiphora.

On the other hand, some disadvantages of STI were reported such as damage to the nasal mucosa or inferior meatus or false passage formation which creates an abnormal passage from canaliculus to nasolacrimal duct, causing failure of the procedures and nasolacrimal duct (NLD) restenosis^8,9^. However, the incidence or management of false passages in LDS has not been examined in previous studies. Using dacryoendoscopy which can explore the inner cavity of the NLD in real time, it is possible to reduce the incidence of false passage by decreasing iatrogenic injury, and diagnose the pre-existing false passage so that proper treatment could be applied. As previous studies on false passage detected in dacryoendoscopy is lack so far, this study is aimed to identify the prevalence and clinical outcomes of patients with false passage in order to suggest the management of them.

## Results

In total, 830 NDO cases underwent STI with dacryoendoscopy. The prevalence of false passages was 2.3%, detected in 19 out of 830 cases. False passages were detected more in women, with an average age of 59.6 ± 4.4 years. Previous history of probing or STI was found in 17 cases (89.4%). They were all performed the lacrimal procedures without dacryoendoscopy. Preoperative average Munk's score was 4.3 ± 0.2, and TMH was measured average 470.4 ± 59.1 μm. The success rate of the total patients with false passage was 73.7%, 14 out of 19 cases (Table 1).

**Table 1:** Demographic characteristics of patients with false passage in dacryoendoscopy (n = 19).

Factor	Data (n = 19, %)
Male: Female (%)	0: 19 (0: 100)
Age (years)	59.6 ± 4.4
Duration of symptom (year)	4.5 ± 1.5
Right eye: Left eye (%)	12: 7 (63.2: 36.8)
Treatment history, % (no. of cases)	89.4 (17/19)
Munk’s score (0–5)	4.3 ± 0.2
Tear meniscus height (μm)	470.4 ± 59.1
Irrigation test, pass: not pass (%)	9: 10 (47.4: 52.6)
Structural change: Secretory change (%)	12: 7 (63.2: 36.8)
Success rate, % (no. of cases)	73.7 (14/19)

Data are mean ± SD unless otherwise stated.

*SD* standard deviation.

**Figure 1:**
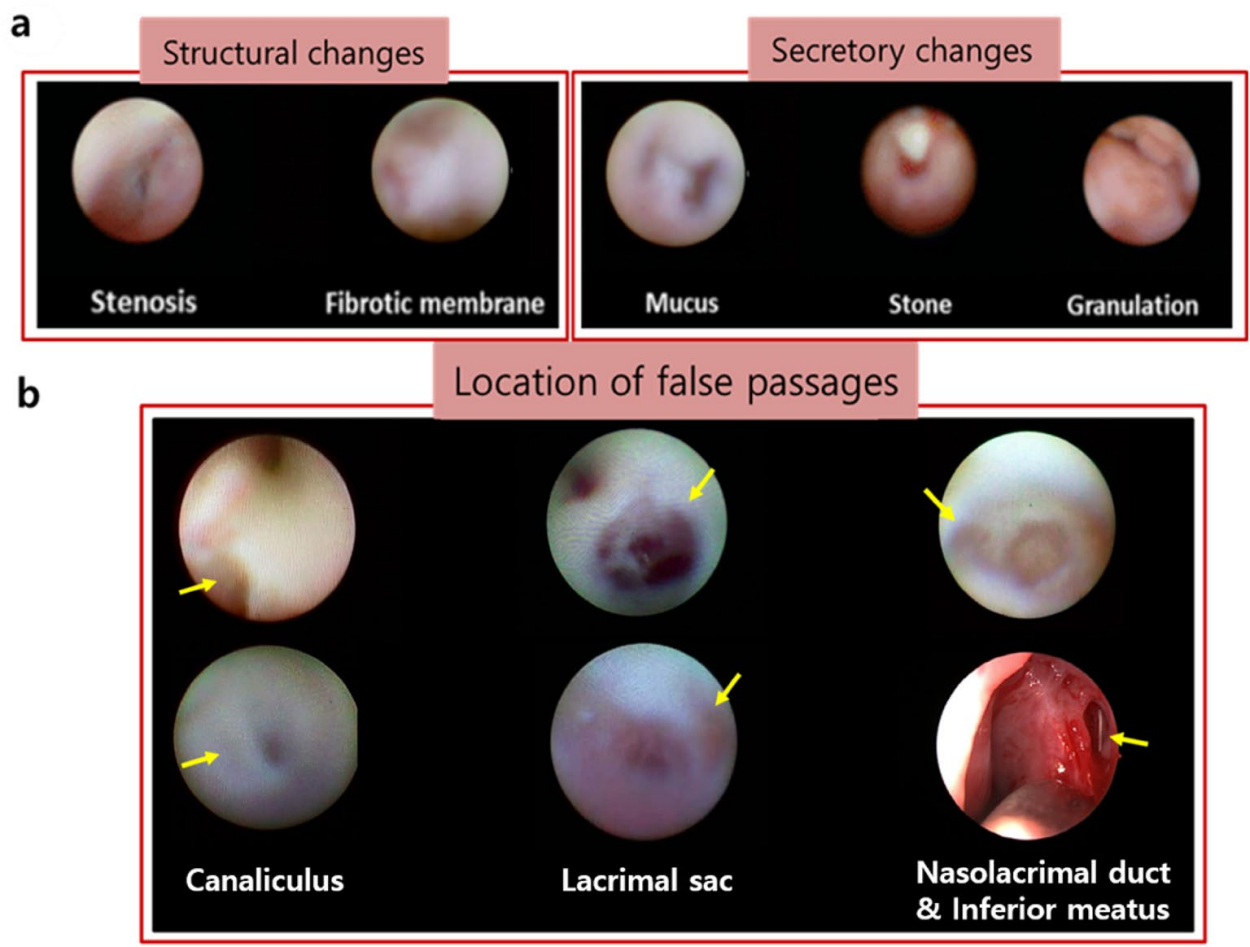
Classification of dacryoedoscopic findings. (**a**) Dacryoendoscopic findings were categorized with structural changes and secretory changes. Structural changes included stenosis and fibrotic membrane, while secretory changes included mucus, stone, and granulation. (**b**) Cases presenting false passages in various locations; canaliculus, lacrimal sac and nasolacrimal duct and inferior meatus. Yellow arrows indicates the false passages beside the normal path.

False passages were observed in canaliculus in 6 cases (31.6%), in lacrimal sac in 6 cases (31.6%), in nasolacrimal duct in 7 cases (36.8%). Dacryoendoscopic findings include structural changes in 12 cases (63.2%), in which 8 cases (42.1%) for stenosis, and 4 cases (21.1%) for fibrotic membrane. The secretory changes were also accompanied in total 7 cases (36.8%), in which 3 cases (15.8%) with mucus, 2 cases (10.5%) with stone, and 2 cases (10.5%) with granulation (Fig. 1). Overall, when false passages were found, they were more often accompanied with structural changes than secretory changes, regrading dacryoendoscopic findings (Table 2).

**Table 2:** Dacryoendoscopic findings in the patients with false passage according to the location and type of obstruction (n = 19).

Factor		Data (n = 19, %)
**Location of obstruction**		
Canaliculus		6 (31.6)
Lacrimal sac		6 (31.6)
Nasolacrimal duct		7 (36.8)
Total		19 (100)
**Type of obstruction**		
Structural change	Stenosis	8 (42.1)
	Fibrotic membrane	4 (21.1)
	Total	12 (63.2)
Secretory change	Mucus	3 (15.8)
	Stone	2 (10.5)
	Granulation	2 (10.5)
	Total	7 (36.8)

**Figure 2:**
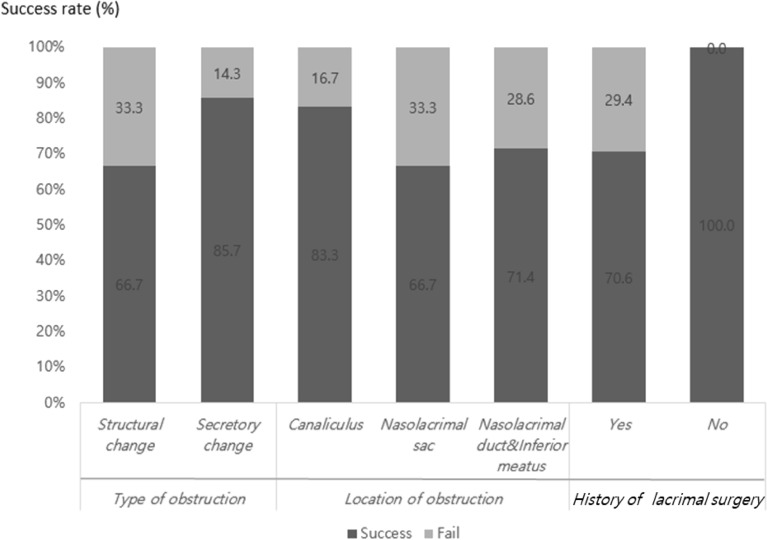
Success rate according to the type and location of obstruction, and previous conventional STI history. Structural change group showed lower success rate than secretory change group. The success rate was the highest in canaliculus group. If the patient had treatment history of probing or STI, the success rate decreased.

The success rate of recanalization of LDS using dacryoendoscopy in the cases in which the false passage was found was investigated according to the dacrycoendoscopic findings (structural change or secretory change), the location of the false passage (canaliculus, lacrimal sac, or nasolacrimal duct and inferior meatus), and the previous laccrimal treatment history (presence or absence). The success rates according to the dacryoendoscopic findings were 6 out of 7 cases (85.7%) with secretory change, and 8 out of 12 cases (66.7%) with structural changes, so the former was higher than the latter. The success rates based on location of the false passage were identified as 5 out of 6 cases (83.3%), 4 of 6 cases (66.7%), and 5 of 7 cases (71.4%), in the canaliculus, lacrimal sac, and nasolacrimal duct, respectively. If there was a previous lacrimal treatment history such as probing or STI, the success rate was 12 out of 17 cases (70.6%), which was lower than the NDO patients without past treatment history (Fig. 2).

## Discussion

Attempts to identify and treat the LDS obstruction, from the punctum to the valve of Hasner, began in 1979 with the study of Cohen et al.^12^, but were not practical at that time. With the development of high resolution microscopic technology, current dacryoendoscopy appeared. According to previous studies regarding the clinical application of dacryoendoscopy, Haefliger and Piffaretti^6^ applied it as a treatment of primary NDO, and introduced it as a proper method to remove lacrimal stone and fibrotic tissue located in the lacrimal duct during surgery. Sasaki et al.^5^ used dacryoendoscopy and nasal endoscope for epiphora patients with NDO, and reported 87.5% as a success rate of surgery during 6 month to 2 year post operative follow-up period. The development of dacryoendoscopy made it possible to visualize inside the nasolacrimal duct and observe the cause of the obstruction of the LDS which could not be detected by DCG, thus leading to make proper management simultaneously.

In order to treat NDO patients, recanalization of LDS through original passage is crucial for success avoiding the false passage formation, as false passage would lead to failure of tear drainage and aggravation of LDS obstruction. We tried to identify and treat the false passages using appropriate technique with dacryoendoscopy. The prevalence of false passage was 2.3% (19 out of 830 cases), and the success rate of the surgery was 73.7% (14 out of 19 cases), which was lower than the previous success rate of ranging from 84.2 to 87.5%^5,7^. As Lee and Lew^7^ suggested, dacryoendoscopic findings revealed that obstruction in LDS could be caused by secretory substances such as mucus and stone as well as structural changes like fibrotic membrane and stenosis. In this study, dacryoendoscopy could be used to categorize the NDO patients on the basis of obstruction type (secretory changes or structural changes) of LDS to allow precise determination of the treatment course. When demonstrating success rate according to dacryoendoscopic findings with structural changes and secretory changes, the success rate was 66.7% (8 out of 12 cases) in structural change, and 85.7% (6 out of 9 cases) in secretory change. This rate was lower than previous study, which stated the success rate as 79.6% in structural change, and 95.3% in secretory change.^7^ However, all patients could be treated successfully even the false passages were found if the patient had no previous lacrimal surgical history, while the success rate decreased to 70.6% in the patients previously treated with conventional silicone tube intubation of LDS. Thus, more attention should be paid at recanalization with patients managed with traditional silicone tube intubation, since false passages could be created. As silicone tube intubation technique is conventionally performed without visualization of LDS, stenosis of the LDS can cause the resistance of advancement and mislead the passages of LDS. Also, the fact that the false passages were accompanied more often in structural changes such as stenosis or fibrotic membrane shows that direct visualizationn during recanalization of LDS may be the key to prevent and manage the false passages in LDS.

Furthermore, the prevalence of false passage accompanied with structural changes was 63.2%, which was higher than with secretory changes of which the prevalence was 36.8%. Previous treatment history could increase the possibility of making structural or secretory changes in the LDS, and especially it seems likely to cause structural changes such as stenosis or fibrotic membrane. When put it together, if there is a history of lacrimal surgery, there could be a high probability of false passages accompanied with structural changes, which can also affect the success rate of surgery. However, as 2 cases showed false passages without previous treatment history, further cytology- and tissue biopsy–based analyses of the LDS are anticipated to investigate the mechanism of false passage formation. Also, we found it that false passages could occur anywhere in LDS, which requires continuous attentions during the lacrimal surgery.

In the cases of false passages, the operation was carried out checking out the true passage through fluid irrigation. When irrigating the fluid, the true passage is expandable like fish mouth, but the false passage is less elastic to the fluid pressure. We also took the advantage of used the previously intubated silicone tube as a reference, recessing back and forth along the silicone tube track, distinguishing false passages from the normal path. Lastly, at the end of recanalization, inferior meatus opening was the landmark to confirm the true passage of the LDS outlet with nasal endoscope to ensure the accurate paths. This study is the first research to investigate the false passages found on dacryoendoscope. And further researches on the natural course and long-term therapeutic outcomes of NDO patients with false passages compared with NDO patients without false passages would be needed. However, as this study was done with small number of samples, it is likely that studies will be anticipated in larger sample numbers in relation to false passages in the future.

In conclusion, dacryoendoscopy enables real-time observation of the lumen of the LDS, thus facilitating treatment of pathological lesions including false passages. With this technique, we are better able to perform customized treatment of patients with false passages, with a safer and more effective results leading to the success of dacryoendosopy guided STI in NDO patients.

## Methods

We retrospectively reviewed the medical records of 830 cases (570 patients) who visited the CHA Bundang Medical Center in Seongnam, South Korea, with epiphora as a chief complaint and underwent transcanalicular dacryoendoscopy with silicone tube intubation from March 2016 to December 2020. False passage was observed in 19 of 830 (2.3%) cases. Patients with epiphora were diagnosed with NDO based upon the lacrimal irrigation, tear meniscus height (TMH), and DCG examination. For lacrimal irrigation test, blunt 23-gauge irrigation needle was connected to 2 mL syringe containing normal saline and inserted into canaliculus through punctum. The results of the test were classified into 'Pass’ and ‘Not pass’. TMH was measured with anterior segment optimal coherent tomography (AS-OCT)^10,11^. DCG was performed to evaluate the obstructed site, using a water-soluble contrast agent, lohexol (Bonorex®; Central Medical Service, Seoul, South Korea).

All procedures were performed by a single surgeon (H.L.). Customized sheath made with an 18-G intravenous catheter or dilator was used to recanalize the lacrimal passage. Then moxifloxacin ophthalmic solution 0.5% (Vigamox; Alcon, TX, USA) and fluorometholone ophthalmic solution 0.1% (Flumetholon; Santen, Osaka, Japan) were irrigated through the LDS. A silicone tube with a diameter of 0.94 mm (Yoowon Meditec, Seoul, South Korea) was inserted through the sheath under visual guidance. The sheath and the tube were retrieved, and both ends of the tube were locked and stabilized under the inferior turbinate. Dacryoendoscope (FT-203F; Fibertech Co., Tokyo, Japan) had the following specifications: probe outer diameter, 0.9 mm; field of view, 70°; number of image elements, 6000; and observation depth, 1–10 mm. The probe of the endoscope was bent at a 27° angle, 1 cm from its tip. A sheath (angiocatheter, 0.92 mm; Daewon, Seoul, South Korea) was wrapped around the probe for covering and dilation of the lumen, with back and forth movement possible inside LDS.

The dacryoendoscope was moved slowly toward the canaliculus and forward gently to the lacrimal sac and nasolacrimal duct. The level of LDS was diagnosed based on the findings of DCG, the dacryoendoscope view inside the LDS, and the nasal endoscopic view confirmed at the end when the dacryoendoscope passed through the LDS. Saline was injected through the water channel for clear viewing of the lumen. The type of obstruction was defined according to dacryoendoscopic findings for the interior of the occluded area from the canaliculus to the inferior meatus, and classified into two groups: structural change group and secretory group. The structural change group included fibrotic membrane and stenosis. The secretory change group included mucus, stones, and granulation (Fig. 1a). The location of the false passage was classified into canaliculus, lacrimal sac, nasolacrmial duct and inferior meatus (Fig. 1b). When false passages were found, we expanded the lacrimal duct lumen by irrigation pressure, then distinguished the opening of the normal path from the false passage. Additionally, the recanalized nasolacrimal duct was verified by the opening through the inferior meatus observed by nasal endoscopy. Otherwise the path was researched and confirmed at the inferior meatus opening to the end. If there was a formerly intubated silicone tube track, we considered it as a reference path.

Outpatient follow-up was done on one week after surgery and one month interval afterwards. Patients’ subjective symptom relief was examined, and measurement of TMH and irrigation test were repeated. Success of the surgery was defined when the epiphora symptoms was relieved leading to decreased Munk’s score, “Passed” at the irrigation test, or TMH was < 300 μm. Statistical analysis was performed with Fisher’s exact test using SPSS Statistics version 25.0 (IBM Corp., Armonk, NY, USA), and defined as significant when the p value was < 0.05. This study and data collection protocol were approved by the Institutional Review Board of CHA Bundang Medical Center (IRB file number: 2022-04-055), and our study design adhered to the tenets of the Declaration of Helsinki. Informed consent was obtained from each subject before enrollment.

## Data Availability

The datasets used and/or analysed during the current study available from the corresponding author on reasonable request.
